# Supplementary material for: Causal Relationship between Aging and Anorexia Nervosa: A White-Matter-Microstructure-Mediated Mendelian Randomization Analysis
Source: Biomedicines. 2024 Aug 16;12(8):1874. doi: 10.3390/biomedicines12081874 (PMC11351342; doi:10.3390/biomedicines12081874)
Supplement: Supplementary file 1 [file biomedicines-12-01874-s001.zip › Supplementary Figure/Figure S4/Singe SNP plot of MR analysis for TL to ubm-b-1532.pdf]

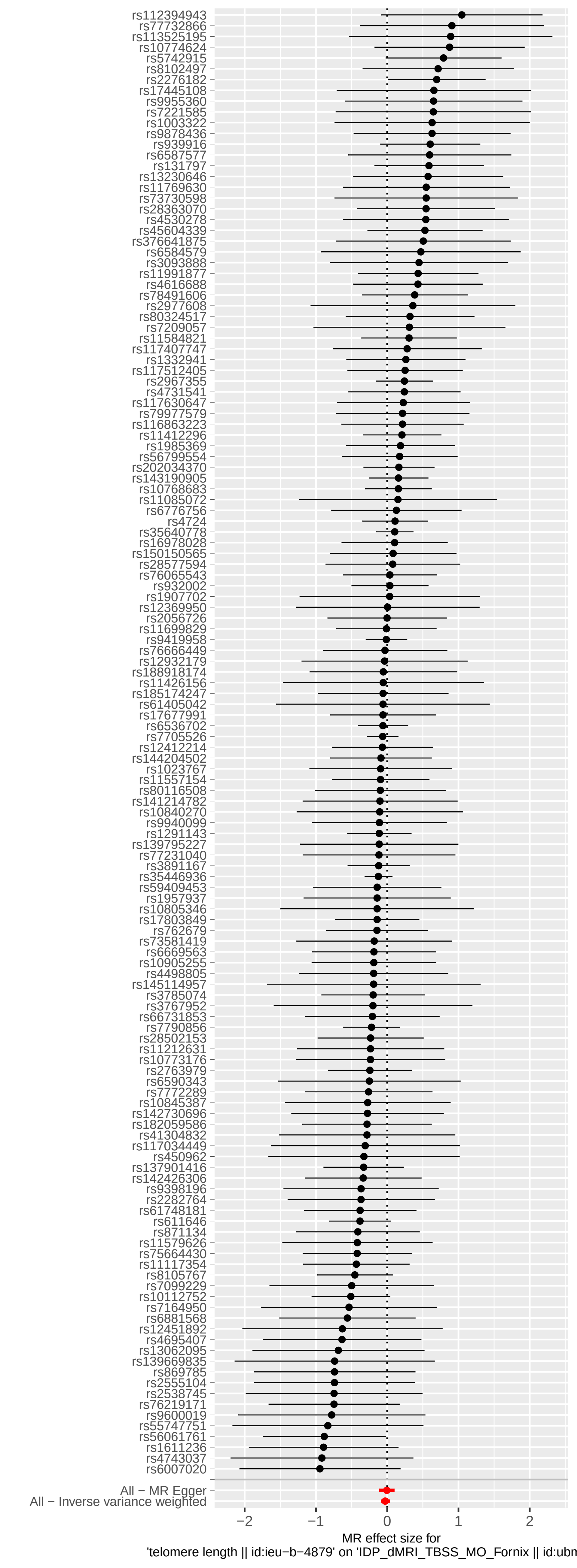
rs112394943
rs77732866
rs113525195
rs10774624
rs5742915
rs8102497
rs2276182
rs17445108
rs9955360
rs7221585
rs1003322
rs9878436
rs939916
rs6587577
rs131797
rs13230646
rs11769630
rs73730598
rs28363070
rs4530278
rs45604339
rs376641875
rs6584579
rs3093888
rs11991877
rs4616688
rs78491606
rs2977608
rs80324517
rs7209057
rs11584821
rs117407747
rs1332941
rs117512405
rs2967355
rs4731541
rs117630647
rs79977579
rs116863223
rs11412296
rs1985369
rs56799554
rs202034370
rs143190905
rs10768683
rs11085072
rs6776756
rs4724
rs35640778
rs16978028
rs150150565
rs28577594
rs76065543
rs932002
rs1907702
rs12369950
rs2056726
rs11699829
rs9419958
rs76666449
rs12932179
rs188918174
rs11426156
rs185174247
rs61405042
rs17677991
rs6536702
rs7705526
rs12412214
rs144204502
rs1023767
rs11557154
rs80116508
rs141214782
rs10840270
rs9940099
rs1291143
rs139795227
rs77231040
rs3891167
rs35446936
rs59409453
rs1957937
rs10805346
rs17803849
rs762679
rs73581419
rs6669563
rs10905255
rs4498805
rs145114957
rs3785074
rs3767952
rs66731853
rs7790856
rs28502153
rs11212631
rs10773176
rs2763979
rs6590343
rs7772289
rs10845387
rs142730696
rs182059586
rs41304832
rs117034449
rs450962
rs137901416
rs142426306
rs9398196
rs2282764
rs61748181
rs611646
rs871134
rs11579626
rs75664430
rs11117354
rs8105767
rs7099229
rs10112752
rs7164950
rs6881568
rs12451892
rs4695407
rs13062095
rs139669835
rs869785
rs2555104
rs2538745
rs76219171
rs9600019
rs55747751
rs56061761
rs1611236
rs4743037
rs6007020
All − MR Egger
All − Inverse variance weighted
−2
−1
0
1
2
MR effect size for
'telomere length || id:ieu−b−4879' on 'IDP_dMRI_TBSS_MO_Fornix || id:ubm
